# Therapeutic strategies for stricturing Crohn’s disease in childhood: a systematic review

**DOI:** 10.1007/s00383-020-04848-0

**Published:** 2021-01-25

**Authors:** Jonathan J. Neville, Alexander Macdonald, John Fell, Muhammad Choudhry, Munther Haddad

**Affiliations:** 1 Department of Paediatric Surgery, Chelsea and Westminster Hospital, London, SW10 9NH UK; 2 Department of Paediatric Gastroenterology, Chelsea and Westminster Hospital, London, UK

**Keywords:** Pediatric surgery, Strictures, Crohn’s disease, Endoscopy, Stricturoplasty

## Abstract

**Purpose:**

Childhood stricturing Crohn’s disease (CD) has significant morbidity. Interventions including resection, stricturoplasty and endoscopic balloon dilatation (EBD) are often required. Optimal intervention modality and timing, and use of adjuvant medical therapies, remains unclear. We aim to review the therapies used in paediatric stricturing CD.

**Methods:**

A systematic review in accordance with PRISMA was performed (PROSPERO: CRD42020164464). Demographics, stricture features, interventions and outcomes were extracted.

**Results:**

Fourteen studies were selected, including 177 patients (183 strictures). Strictures presented at 40.6 months (range 14–108) following CD diagnosis. Medical therapy was used in 142 patients for an average of 20.4 months (2–36), with a complete response in 11 (8%). Interventions were undertaken in 138 patients: 53 (38%) resections, 39 (28%) stricturoplasties, and 17 (12%) EBD. Complications occurred in 11% of resections, versus 15% stricturoplasties, versus 6% EBD (*p* = 0.223). At a median follow-up of 1.9 years (interquartile range 1.2–2.4) pooled stricture recurrence was 22%. Resection had 9% recurrence, versus 38% stricturoplasty, versus 47% EBD (*p* < 0.001).

**Conclusions:**

Resection is associated with a low incidence of recurrence and complications. There remains a paucity of evidence regarding adjuvant medical therapy and the role of EBD. We propose a minimum reported dataset for interventions in paediatric stricturing CD.

## Introduction

Crohn’s disease (CD) is a chronic, relapsing–remitting inflammatory disease of the gastrointestinal tract. It presents in childhood and adolescence in up to 25% of cases and has a significant impact on development and quality of life [1]. Incidence of paediatric CD is 2.5–11.4 per 100,000 worldwide and increasing [2, 3]. Management of the disease in children aims to induce and maintain remission, prevent and treat complications, and—unique to the paediatric population—facilitate normal growth [3].

Paediatric CD may be described by the Paris classification, incorporating age at diagnosis, disease location, disease behaviour (B), and its effect on growth [1]. In children, the behavioural phenotypes include non-stricturing and non-penetrating disease (B1), stricturing (B2), penetrating (B3), and both stricturing and penetrating concurrently or at different points in time (B2B3). Complicated CD behaviour (B2 and B3) occurs in ~ 12–20% of children at diagnosis, increasing to 20–60% after 3–7 years [4–6]. Approximately 25% of children with CD will develop stricturing disease (B2) at some point [4]. Complicated CD often requires intervention, and estimated lifetime risk of surgery in this patient group is 50–90% [7, 8].

Medical therapies are used to induce or maintain remission in paediatric CD [3, 9]. However, symptomatic stricturing disease, refractory to medical management, will often require non-medical intervention [10]. Alternatives to the surgical resection of a strictured segment of bowel, include stricturoplasty and endoscopic balloon dilatation (EBD), which treat strictures without compromising bowel length. These strategies may be preferable to surgical resection, as strictures tend to recur postoperatively regardless of treatment modality [8, 10, 11]. Choice of intervention is a balance between the efficacy of treatment and the potential for complications. As such, there remains debate surrounding the optimal timing and modality of non-medical intervention in stricturing CD, and the role of adjuvant medical therapy in delaying or preventing surgery.

The primary aim of this systematic review was to evaluate the current therapeutic strategies used in paediatric patients with stricturing CD. We sought to investigate the efficacy of each treatment strategy in preventing stricture recurrence and compare complication rates. The secondary aim of this review was to evaluate the quality of the current evidence base, to provide recommendations and future directions for research.

## Materials and methods

A systematic review of the literature was performed according to the preferred reporting items for systematic reviews and meta-analysis (PRISMA) guidelines. The study protocol was specified in advance and stored on PROSPERO (CRD42020164464).

### Data sources

An electronic search was performed of MEDLINE, SCOPUS and the Cochrane Library databases from inception to January 2020. The following search string was used: (“p?ediatric” OR “child*”) AND (“Crohn’s disease” OR “Crohn’s” OR “inflammatory bowel disease”) AND (“strictur*” OR “stenosis” OR “fibrosis”) AND (“biologics”, OR “anti-TNF”, OR “infliximab”, OR “anti-fibrotic”, OR “surgery”, OR “colectomy”, OR “resect*”, OR “stricturoplasty”, OR “endoscopic balloon dilatation”). Reference lists of selected articles were also searched for relevant studies.

### Study selection

Studies reporting the outcomes following medical and/or surgical management of stricturing Crohn’s disease, in patients under the age of 18 years, were selected for inclusion. Review articles, clinical guidelines, and studies not published in English were excluded. Studies which failed to differentiate adult and paediatric patients, or between inflammatory bowel diseases and Crohn’s disease, were also excluded.

### Assessment of study quality

Study quality was assessed according to a previously published approach for similar quantitative and qualitative research [12]. Articles were scored on a three-point ordinal scale across a series of assessment criteria (zero if criteria not met; one if criteria partially met; and two if criteria definitely met). All studies were scored on nine criteria. If the criterion was not applicable to the study, the total score was reduced by two and no score was given. For the purposes of comparison, each total score has been presented as a percentage of the maximum possible score. No studies were excluded based on quality.

### Data extraction and synthesis

The primary outcome was a recurrence of stricturing disease requiring further treatment (medical or surgical). The secondary outcomes investigated were: the achievement of disease remission, complications of treatment and mortality. Patient demographics, features of the stricturing disease and information regarding initial treatment modality were also extracted.

Statistical analysis was conducted using SPSS version 24 (IBM). Data is quoted as mean (range) or median (interquartile range). The Chi-squared test was used to compare categorical variables. Statistical significance was set at p < 0.05.

## Results

### Included studies

**Fig. 1:**
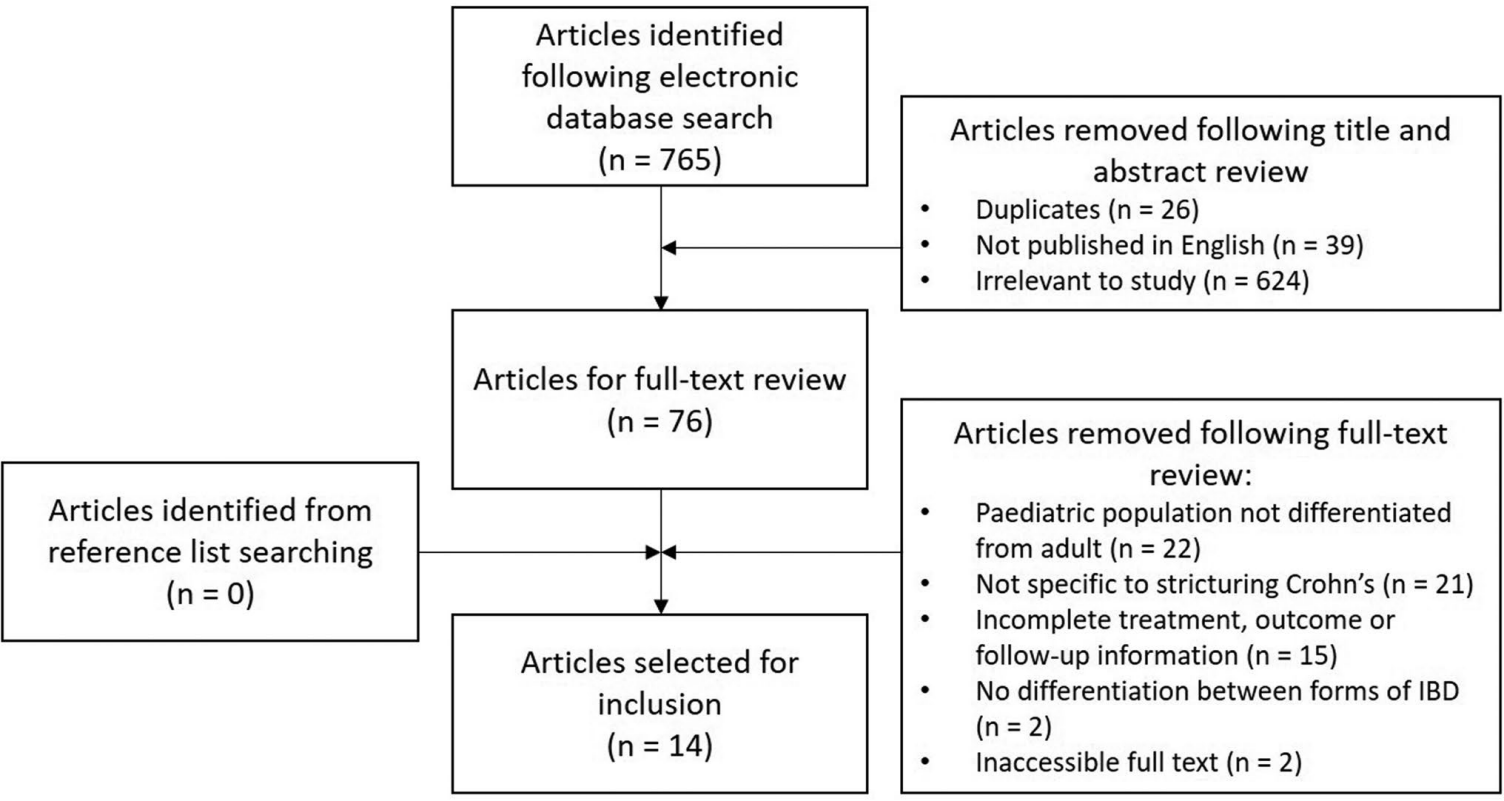
PRISMA flowchart highlighting the results of the literature search

Electronic database searching identified 765 articles (Fig. 1). Duplicates and articles not published in English were removed. After title and abstract screening, 76 full-text articles were reviewed, and 14 studies were selected for inclusion (Table 1). These studies comprised of one randomised control trial, three retrospective cohort studies, one prospective observational study, seven case series, and two case reports. One study was multicentre. Eleven studies were from specialist paediatric centres and three studies were mixed paediatric and adult. A total of 177 patients were analysed, 72 (41%) were female. Mean ages reported across the 14 studies ranged from 10 to 17 years. Patient co-morbidities were unspecified in eight studies. Two patients had trisomy 21 and four patients had no co-morbidities.

**Table 1:** Summary of articles selected for inclusion

Author	Year	Design	Location	Cohort size	Frequency female (%)	Mean age (range)	Intervention(s)	Mean follow-up (years)	Quality score (%)
Aloi et al*.* [15]	2013	SS, retrospective cohort	Italy	36	12 (33.3)	14.7 (7.3–20.2)	Medical 32 Surgery NS 4	2	11 (68.8)
Bamford et al*.* [8]	2014	SS, retrospective cohort	UK	26	13 (50.0)	15.6 (7.2–19.4)	Stricturoplasty 13 Resection 13	5.6	12 (75.0)
Burgess et al*.* [31]	2019	SS, case series	UK	2	1 (50.0)	14 (13–15)	Gastroduodenostomy 2	2.15	12 (66.7)
Abriola et al*.* [26]	2003	SS, case series	Italy	5	3 (60.0)	16 (14–20)	Stricturoplasty 5	1.8	7 (43.8)
Di Nardo et al*.* [24]	2010	SS, randomised control trial	Italy	29	13 (44.8)	14.5 (9–17.5)	EBD + intralesional steroid injection 15 EBD 14	1	15 (83.3)
Dutta et al*.* [32]	2003	SS, case series	USA	15	NS	14 (9–17)	Resection 15	4	7 (43.8)
Inoue et al*.* [33]	2013	SS, case report	Japan	1	0 (0)	14	Resection 1	1.5	7 (58.3)
Ko et al*.* [34]	2011	SS, case series	Korea	2	0 (0)	15	EBD 2	0.9	12 (75.0)
Lawal et al*.* [35]	2010	SS, case series	USA	2	1 (50.0)	17	Diverting colostomy 1 Diverting ileostomy 1	1.1	6 (42.9)
Lourenco et al*.* [16]	2016	SS, case series	Portugal	4	2 (50.0)	11.4 (7.9–15.1)	Resection 4	1.9	11 (68.8)
Oliva et al*.* [30]	1994	SS, case series	USA	8	6 (75.0)	15.8 (9.9–18.5)	Stricturoplasty 2 Stricturoplasty + resection 6	1.5	6 (37.5)
Rahhal et al*.* [36]	2007	SS, case report	USA	1	1 (100.0)	10	EBD 1	0.25	7 (58.3)
Romeo et al*.* [7]	2012	MS, retrospective cohort	Italy	39	16 (41.0)	11.8 (4–17)	Resection 20 Stricturoplasty 19	6.9	12 (75.0)
Salvatore et al*.* [37]	2000	SS, prospective observational	UK	7	4 (57.1)	15.3 (13.6–17.9)	Medical 7	2.5	11 (68.8)

*SS* single site, *MS* multi-site, *NS* not specified, *EBD* endoscopic balloon dilatation

Quality assessment was undertaken in all studies (Table 1). Mean study quality was 62% and ranged from 38 to 83%. In particular, articles scored poorly on the discussion of exclusion criteria, statistical analysis methods, and study limitations.

### Indications for intervention

Time from the initial diagnosis to the development of a CD stricture was unspecified in seven studies (totalling 122 patients). Stricturing disease was identified at a presentation in 18 patients. Of the remaining 37 patients, the mean time from the initial diagnosis to the development of a stricture was 40.6 (14–108) months. The commonest clinical features reported were abdominal pain (57%), growth failure (12%) and vomiting (9%) (Table 2). Pre-intervention imaging modalities were specified in 100 patients: 71 underwent a magnetic resonance imaging scan, 55 underwent a contrast study, and three underwent endoscopy.

**Table 2:** Clinical features (*n* = 177)

Clinical Features	Frequency of patients reporting feature (%)
Abdominal pain	100 (56.5)
Growth failure/weight loss	22 (12.5)
Vomiting	16 (9.0)
Diarrhoea	9 (5.1)
Constipation	8 (4.5)
Obstructive symptoms^a^	7 (4.0)
Rectal pain	1 (0.6)
Dysphagia and odynophagia	1 (0.6)
Not specified	35 (19.8)

^a^Referring to symptoms of intestinal obstruction not otherwise specified in included articles

A total of 183 individual strictures were reported and treated, corresponding to: 42% ileocolic, 36% ileal, 13% upper-gastrointestinal, 4% colonic, 1% rectal and 0.5% oesophageal. One study (eight patients) did not specify the stricture location. Twelve strictures were anastomotic. The nature of the stricture was reported in 62 cases. Inflammatory strictures occurred in 26 cases, and 36 strictures were classified as fibrotic, or mixed fibrotic and inflammatory. Five studies specified stricture length, and mean length varied from 1.5 to 35 cm and ranged between 1 and 80 cm.

### Types of intervention

Medical therapy was used to treat stricturing disease in 142 patients and combination therapy was commonplace. The commonest medical therapies used were oral/intravenous steroids (47%), 5-ASA (32%), immunomodulators (30%) and biologics (14%). Infliximab was used in six patients, adalimumab in two, and the biologic used was unspecified in 12. Thalidomide was used as a rescue therapy in one patient.

A total of 138 patients underwent some form of intervention (Table 3). These included 53 resections (38%), 39 stricturoplasties (28%), 17 EBD (12%), 15 EBD with intra-lesional steroid injections (11%), and six stricturoplasties with concomitant resections (4%). One patient underwent a diverting colostomy, another a diverting ileostomy, and two patients had gastroduodenostomies. Intervention type was unspecified in four cases. Of these 138 procedures, 106 followed medical treatment. Only four studies specified the duration of medical treatment prior to intervention; the mean duration was 1.7 years but ranged from eight weeks to 3 years.

**Table 3:** Summary of primary and secondary outcomes. ‘Surgery other’ includes diverting ileostomy, colostomy and gastroduodenostomy

	EBD (*n* = 17)	Stricturoplasty (*n* = 39)	Surgical resection (*n* = 53)	Surgery other/NS (*n* = 8)	*P*-value*
Recurrence of stricture requiring further treatment (%)	8 (47.1)	15 (38.5)	5 (9.4)	2 (25.0)	< *0.001*
Complications of the procedure (%)					
Total	1 (5.9)	6 (15.4)	6 (11.3)	2 (25.0)	0.223
Abscess	0 (0)	2 (5.1)	1 (1.9)	0 (0)	
Anastomotic leak	0 (0)	1 (2.6)	2 (3.8)	0 (0)	
Fistula	1 (5.9)	0 (0)	0 (0)	1 (12.5)	
LRTI	0 (0)	1 (2.6)	1 (1.9)	0 (0)	
Return to theatre	0 (0)	1 (2.6)	0 (0)	0 (0)	
Ileus	0 (0)	1 (2.6)	1 (1.9)	0 (0)	
Adhesiolysis	0 (0)	0 (0)	1 (1.9)	0 (0)	
Wound infection	0 (0)	0 (0)	0 (0)	1 (12.5)	

*NS* not specified, *EBD* endoscopic balloon dilatation, *LOS* length of stay, *IQR* interquartile range, *LRTI* lower respiratory tract infection

*Chi-squared test

### Outcomes

Complete response of the stricturing CD to medical therapy was only seen in 11 patients (8%). Incomplete or no response was seen in the remaining 131 (92%) medically treated patients. Only 36 patients received medical therapy alone, in which group the recurrence rate of stricturing disease was 39%.

Pooled relapse rate was 22% after intervention. Thirty-nine patients required further interventions, corresponding to a total of 82 subsequent procedures over a median 1.9 (1.2–2.4) year follow-up period. Resection was significantly associated with a lower rate of stricture recurrence when compared to EBD and stricturoplasty (*p* < 0.001).

One study reported a change in *Z*-score for weight. In eight patients following stricturoplasty a 0.5 increase in the *Z*-score mean was observed (*p* = 0.040). Three studies discussed Paediatric Crohn’s Disease Activity Index (PCDAI) scores pre- and post-intervention. Pre-intervention, Aloi et al. cohort of 36 patients had a mean PCDAI score of 31.0 ± 17. This decreased to 30.0 ± 17 in the medically treated group (*n* = 32), but increased to 39.0 ± 14 in the surgically treated group (*n* = 4). However, the difference in post-intervention PCDAI was not significant. Diabriola et al*.* observed a decrease in mean PCDAI from 51.1 to 8.6 at 6 months in five patients treated with stricturoplasty. Similarly, Lourenco et al. reported a fall in mean PCDAI from 35.0 ± 13 to 6.9 ± 8 in four patients treated with surgical resection.

Ten studies reported complications following the intervention. These included: three abscesses; three anastomotic leaks; two fistulae; two lower respiratory tract infections; and two ileus. One patient had to return to theatre for unspecified reasons and a second required adhesiolysis after 8 months. Upon pooling the frequency of complications across resection, stricturoplasty and EBD, no significant difference in complication rate was observed (*p* = 0.223).

## Discussion

Stricturing CD in childhood is a source of major morbidity, and symptomatic disease refractory to medical treatment requires intervention. The optimal timing and modality of intervention, and the role of adjuvant medical therapy, remains unclear. There is a paucity of literature comparing interventions in stricturing CD in the paediatric population.

In this systematic review, strictures were found to recur in 22% of children following intervention; failure to respond to medical therapy was 92%. Decisions regarding the best treatment choice in these patients are a balance between efficacy and definitiveness of treatment, and the potential for complications. We have observed that surgical resection of strictures associates with a significantly decreased rate of recurrence when compared against stricturoplasty and EBD. Complication rates following all evaluated interventions are low and do not differ significantly.

### Indications for intervention

Intervention in paediatric CD is undertaken when medical therapy fails to halt progression, control symptoms, or complications of the disease occur. Strictures in CD often present with the clinical features of gastrointestinal obstruction. In this cohort, children presented with abdominal pain, weight loss and vomiting. Diagnostic imaging modalities include magnetic resonance enterography, contrast studies, ultrasonography and endoscopy. Balloon-assisted endoscopy is also utilised in paediatric patients but was not reported by studies in this review [13].

Both the location and behaviour of child-onset CD have been shown to be dynamic. Disease extension occurs in up to 40% of cases, and 20–60% of patients develop complicated CD over time [4, 6]. Stricturing CD is most common in older children aged > 10 years [14, 15]. Ileocolic disease is most commonly seen in children, and the strictures in this review were most commonly located in the ileocolic or ileal regions [4, 14]. Of the 183 strictures reported in this review, 14% were inflammatory, and 20% were fibrotic or mixed fibrotic and inflammatory. Typically, inflammatory strictures respond better to medical immunomodulatory therapies, whereas fibrotic strictures require non-medical management.

### Interventions

#### Medical therapy

A number of studies suggest that early and prolonged use of immunomodulatory therapies may delay or prevent the formation of strictures, and postpone intervention [16, 17]. There remains debate surrounding the optimum timing and duration of medical treatment necessary to avoid intervention. In this review, the complete response of stricturing CD to medical therapy was seen in 8% of patients, suggesting that in certain cases stricturing disease may be reversed by immunomodulation. Aloi et al. observed a significant association between response to medical therapy and features of active stricture inflammation on imaging, and reported a 35% response rate of stricturing disease to medical therapy at 2 years [15].

In this review, the mean duration of medical therapy was only 1.7 years before escalation, and 77% of interventions followed some form of medical therapy. There are limited details in the literature regarding the rationales for escalation, and so these may differ considerably between the studies analysed here. However, it may be concluded that earlier initiation of medical therapy, or medical treatment in patients with specifically inflammatory strictures, may prevent or reverse stricturing disease.

In this cohort, 14% of patients received biologic therapy. Biologics are effective at inducing and maintaining remission in active CD in children, and treatment with infliximab has been shown to associate with the regression of bowel stenosis in certain patients [3, 18, 19]. A number of studies included in this review were published prior to the era of widespread biologic usage. This may explain the relatively low proportion of patients receiving such treatment prior to non-medical therapies. That, and a focus on surgical treatments and outcomes in the included studies, may result in an underestimation of the benefits of biologic therapy in paediatric stricturing CD.

#### Endoscopic balloon dilatation

EBD is considered the optimal bowel-preserving intervention for short and isolated strictures, and in particular anastomotic strictures and those located at the ileocecal valve [20]. EBD is a useful, low-cost alternative treatment to stricturoplasty and surgical resection. It avoids potential surgical morbidities, such as wound complications, anastomotic leaks, stoma formation and short bowel syndrome. In adults, the complication rate of EBD is 15–20% [20]. However, 30–75% of patients who undergo EBD require surgery within 5 years [20–23].

Strictures that are longer and inflammatory associate with a poorer outcome following EBD [21]. Di Nardo et al. compared EBD with concomitant intralesional steroid injection against EBD with placebo injection, in a randomised control trial [24]. They observed a significantly lower rate of repeat dilatations and subsequent surgery in the EBD with steroid injection group, compared to placebo. A few small studies in adults investigating the efficacy of intralesional steroid injections combined with EBD have reported conflicting outcomes [25]. EBD with steroid injection may be an option for inflammatory strictures in children, but further research is required.

On balance, although complication rates for EBD are low, it appears that it represents a temporalizing measure. There are limited studies with long-term follow-up assessing the efficacy of EBD in children. A large proportion of children who undergo EBD for stricturing CD may require surgery at a later date.

#### Stricturoplasty

Stricturoplasty can be beneficial over bowel resection because it avoids a reduction in bowel length. Complication rates compared to surgical resection are generally low [7, 8, 26]. However, studies directly comparing stricturoplasty to bowel resection in paediatric patients have shown conflicting results.

In a retrospective review of 26 patients, Bamford et al. observed a significantly higher rate of subsequent surgery in those who underwent a stricturoplasty, compared to those who underwent a resection or combined procedure [8]. In contrast, Romeo et al. observed no significant difference in stricture recurrence rate in patients undergoing bowel resection and stricturoplasty [7]. Three patients in the stricturoplasty group developed a recurrence, and all required further endoscopic or surgical treatment. The cohort size in this study was small (20 resections versus 19 stricturoplasties) and it may have been underpowered to detect a significant difference in stricture recurrence. However, all patients with recurrent disease in the stricturoplasty group required further intervention, suggesting that resection may offer a more definitive treatment option.

#### Resection

Bowel resection has the potential to effect definitive treatment of strictures in CD, but also carries the potential for surgical complications. Because of the relapsing–remitting nature of CD, and the increasing risk of disease recurrence with age, there is also the potential for repeated resections resulting in short bowel syndrome.

Studies investigating the long-term outcomes following resection in children report that 14–22% of patients require repeat surgery at 6–10 years [27, 28]. The immediate commencement of medical therapy post-resection associates with a reduction in clinical and surgical recurrence rate [28]. Predictors of a poorer outcome and recurrence include female sex, ileocecal disease, positive resection margins, and the presence of extra-intestinal manifestations [27, 28].

Over time, the number of paediatric and adult patients undergoing resection for CD is falling, and there is a shift from emergency to elective bowel resection [29]. This may be resulting from the more widespread usage of improved medical therapies, alternative surgical treatment options, and multi-disciplinary care. However, in certain patient groups, resection may be superior to stricturoplasty and EBD when combined with adjuvant medical therapy. Definitive management through resection also avoids the morbidities associated with repeated stricturoplasties or balloon dilatations.

### Quality of included studies

Overall, the studies included in this review were of moderate quality. Areas of particular weakness included the discussion of exclusion criteria (if applicable), reporting ethical approval and/or patient consent to participate in the study, explanation of the statistical analyses used, and a discussion of the study limitations and external validity.

Significant heterogeneity in the methodology and reporting of studies included in this systematic review was observed. Patient demographic information often lacked co-morbidities that may be relevant for operative planning and interpretation of outcomes. The indications for intervention and results of pre-intervention imaging studies were infrequently reported. Only one study described participant weights before and after the intervention, making the impact of the intervention on catch-up growth difficult to assess [30].

As such, we have developed a minimum reported dataset for studies investigating the effects of non-medical treatment strategies in paediatric stricturing CD (Table 4). This will improve the quality and comparability of further research. Of those included in this systematic review, no studies met these requirements fully.

**Table 4:** A minimum reported dataset for studies investigating non-medical interventions in paediatric stricturing Crohn’s disease (CD)

Category	Minimum reported dataset
Demographics	Age Gender Co-morbidities Institution service type (paediatric, adult or both)
Crohn’s disease	Date of CD diagnosis CD phenotypic description as per the Paris Classification[1]
Stricture disease	Location of stricture Inflammatory/fibrotic/mixed stricture Length Association with anastomosis Date of diagnosis
Indications for intervention	Rationale for intervention Symptomatology Imaging results Weight
Interventions	Type of intervention Adjuvant medical therapy Timing of intervention
Outcomes	Stricture surgical recurrence (defined as requiring further medical or surgical treatment) Stricture clinical recurrence (defined as causing clinical features) Hospital length of stay post-intervention Complications of intervention Requirement for repeat procedures post-intervention *Z*-score for weight Quality of life assessment (paediatric Crohn’s disease activity index) Mortality

### Limitations

This review has a number of limitations to consider. A number of studies were excluded from inclusion because they were not published in English, paediatric and adult patients were not clearly separated, or subtypes of inflammatory bowel disease were not differentiated. The failure to include these studies weakens the power of this review.

The studies included in this systematic review were small, single centre and mostly retrospective, and all had follow-up periods < 7 years. Smaller case reports and series were not excluded from the study due to the paucity of evidence on the topic. Taken together, this reduces the ability to draw long-term conclusions on the efficacy of each treatment modality; short follow-up periods may underestimate recurrence. There was also significant heterogeneity in the reporting of demographic information, symptomatology, stricturing disease phenotype, and outcome measures following the intervention. Few studies documented pre- and post-intervention PCDAI.

Although the type of medical therapy used was reported in the majority of studies, there was limited information regarding the duration of treatment prior to surgical or endoscopic intervention and the rationale for escalation to non-medical treatments. Few studies reported post-intervention adjuvant medical therapies, and no information was available on medical therapy escalation. This may lead to an overestimation of stricture recurrence in the medically treated cohort.

The inflammatory or fibrotic nature of each stricture was only specified in 34% of cases, and it is probable that the majority of the unspecified strictures were fibrotic in nature. Because of the potential over-representation of fibrotic stricturing disease in the selected studies, it may be that the efficacy of medical therapies is underestimated compared to surgical treatments.

### Implications for surgical practice and future research

Surgical resection appears to offer a definitive treatment strategy, with lower recurrence rates and comparable complication rates compared to stricturoplasty and EBD. However, this review has also highlighted a paucity of prospective studies directly comparing different treatment strategies in childhood stricturing CD with a long follow-up period. This limits the conclusions that can be drawn regarding the long-term efficacy of each intervention and the potential benefits of bowel-preserving treatment strategies. Collaborative, multicentre studies and/or registries comparing the effects of resection, stricturoplasty and EBD on stricture recurrence rate and long-term outcome are required to identify the optimum treatment strategy in these patients. Further studies are also required to investigate the role of medical therapy in postponing intervention, or in preventing recurrence after the intervention.

### Conclusions

In this study, the majority of children failed medical therapy and required intervention. Surgical resection is associated with a low incidence of recurrence and complications, and represents definitive treatment. Further work is required to elucidate the role of medical management in stricturing CD and to compare outcomes following non-medical interventions in prospective trials with long-term follow-up and defined outcome sets.

## Abbreviations

CD: Crohn’s disease
EBD: Endoscopic balloon dilatation
PRISMA: Preferred reporting guidelines for systematic reviews and meta-analyses
SS: Single site
MS: Multi-site
NS: Not specified
LOS: Length of stay
IQR: Interquartile range
LRTI: Lower respiratory tract infection
